# A case of pacemaker implantation in the patient with duplication of the left innominate vein: a case report

**DOI:** 10.1186/s40064-016-2182-9

**Published:** 2016-04-26

**Authors:** Iwanari Kawamura, Rintaro Hojo, Seiji Fukamizu

**Affiliations:** Department of Cardiology, Tokyo Metropolitan Hiroo Hospital, 2-34-10, Ebisu, Shibuya-ku, Tokyo, 150-0013 Japan

**Keywords:** Duplication of the left innominate vein, Retroaortic innominate vein, Pacemaker

## Abstract

**Introduction:**

Duplication of the left innominate vein is a rare systemic venous anomaly defined as the coexistence of a retroaortic innominate vein and a normally positioned left innominate vein. We describe a successful case of pacemaker implantation in a patient with duplication of the left innominate vein via a retroaortic innominate vein.

**Case description:**

A 70-year-old mentally challenged man was admitted to our hospital because of bradycardia and an altered state of consciousness. Electrocardiogram indicated sinus arrest and junctional escape rhythm with a heart rate of 40 beats/min; hence, a pacemaker was implanted. Left subclavian venography showed two vessels that were connected to the superior vena cava: a narrow, normal positioned left innominate vein and a tortuous vein. The normally positioned left innominate vein was too narrow to pass through with a guide wire. Therefore, we chose the tortuous vein for implantation. However, the procedure was difficult because of the vein’s tortuosity. Finally, leads at the right atrium and ventricle were successfully implanted using a steerable stylet. After the procedure, computed tomography showed two innominate veins: a retroaortic innominate vein and narrow left innominate vein that was a duplication of the left innominate vein.

**Discussion and evaluation:**

The exact embryogenesis of retroaortic innominate vein remains unknown and incidence of retroaortic innominate vein is very rare. But for cardiologists performing transvenous pacemaker insertion, the anomalous brachiocephalic vein may cause technical difficulty during a left arm approach.

**Conclusions:**

In cases in which subclavian venography shows a tortuous vein, cardiologists should consider the presence of a retroaortic innominate vein.

## Background

The retroaortic innominate vein (RAIV) is a rare systemic venous anomaly characterized by an abnormal position of the left innominate vein (LIV) behind the ascending aorta. In cases with an RAIV, confluence of the left subclavian and left common jugular veins form the LIV, which then turns inferior, a course that is initially similar to that of a persistent left superior vena cava (PLSVC). After passing the left pulmonary artery anteriorly and before reaching the left atrium (LA), the LIV turns rightward and courses horizontally behind the ascending aorta to reach the superior vena cava (SVC). The RAIV enters the SVC only a short distance above the SVC-right atrium (RA) junction. Another rare anatomic variation is duplication of the RAIV, which is defined as the coexistence of a RAIV and a normally positioned LIV (Hugh et al. 2012; Gerlis 1989).

Most patients with a RAIV have an associated congenital cardiac malformation (Nagashima et al. 2010). The incidence of a RAIV is 0.2–1 % in all cases with a congenital cardiac anatomy, but an isolated RAIV without associated cardiac or arch anomalies is extremely rare (Sivasubramanian et al. 2013; Chen et al. 2005; Gülsün et al. 2005). In the present report, we describe the case of a man with isolated RAIV who underwent pacemaker implantation via a tortuous RAIV.

## Case description

**Fig. 1:**
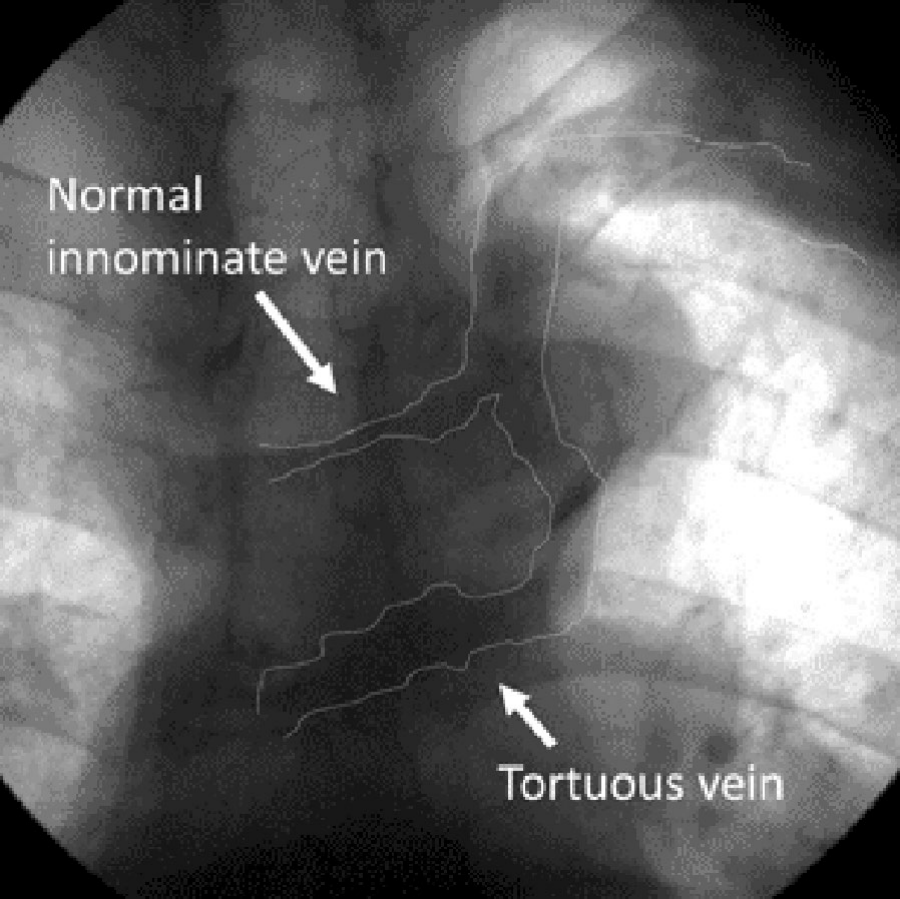
Left subclavian venography showed two vessels which connected to the superior vena cava

**Fig. 2:**
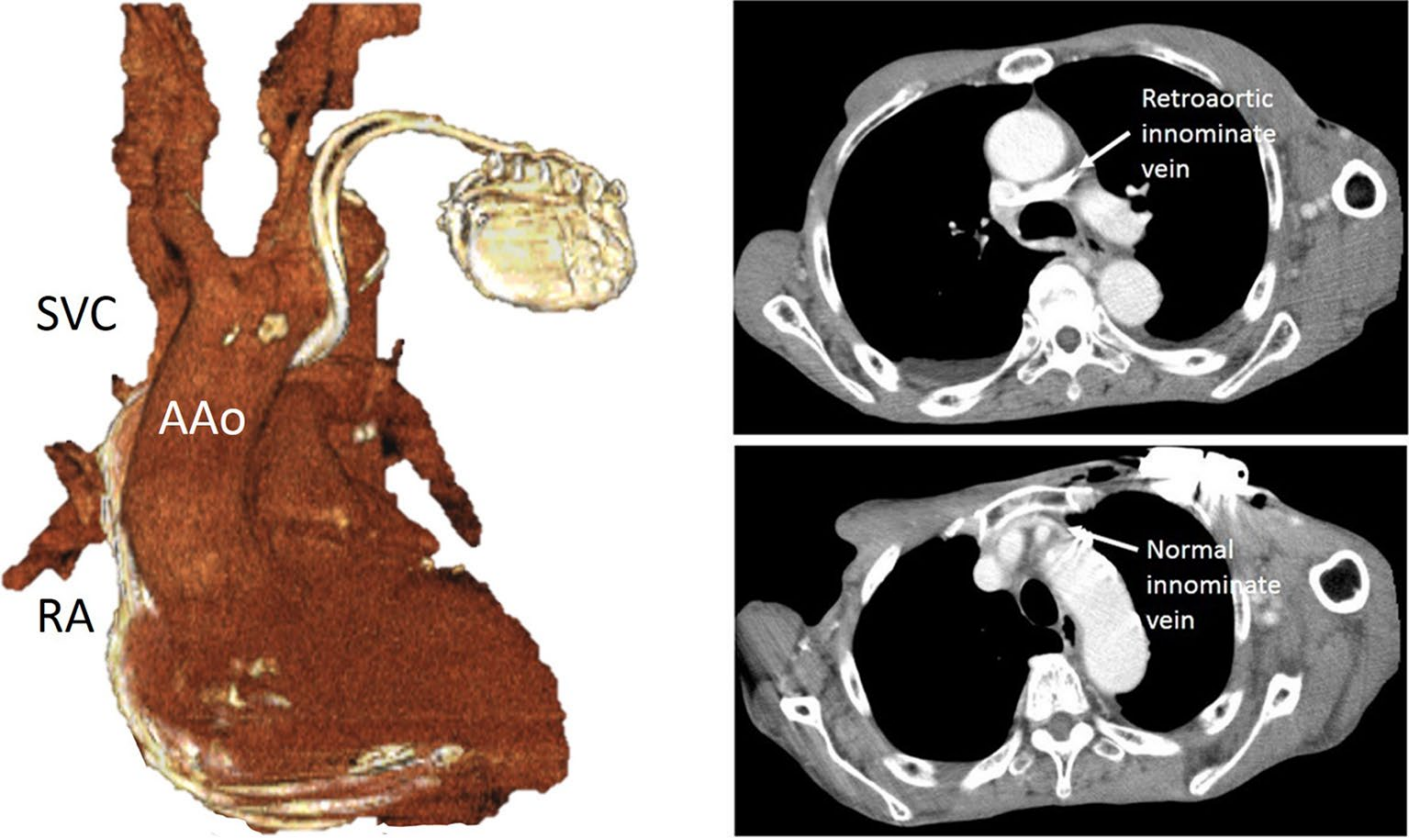
Computed tomography revealed duplication of left innominate vein. There was no other abnormality

A 70-year-old mentally challenged man was admitted to our hospital because of bradycardia and an altered state of consciousness. On the day of hospitalization, he suddenly suffered from disturbance consciousness after breakfast, and his heart rate was 40 beats/min. Electrocardiogram indicated sinus arrest and a junctional escape rhythm; hence, a pacemaker was implanted. There was no congenital heart disease on transthoracic echocardiogram on admission so we had not observed duplication of the LIV before the procedure. Left subclavian venography showed two vessels that were connected to the superior vena cava: a narrow, normally positioned LIV and a tortuous vein (Fig. 1). The tortuous vein turned inferiorly and ran a course similar to that of the PLSVC. Before reaching the LA, the tortuous vein turned rightward and coursed horizontally into the SVC. There was only a short distance from where the RAIV entered the SVC. The normally positioned LIV was too narrow to pass through with a guide wire. Therefore, we chose the tortuous vein for pacemaker implantation. However, the procedure was difficult because of the vein’s tortuosity. Finally, leads at the right atrium and ventricle were successfully implanted using a steerable stylet (Locator™, St. Jude Medical, Inc., St. Paul, MN, USA). After the procedure, computed tomography showed two innominate veins: a RAIV and a narrow LIV that was a duplication of the LIV (Fig. 2).

## Discussion and evaluation

The exact embryogenesis of RAIV remains unknown. Previous reports have proposed the presence of two transverse channels initially in the early embryo, one located superiorly and the other inferiorly. Subsequently, the lower regress and the upper regress become the normal left brachiocephalic vein. The anomalous brachiocephalic vein is therefore thought to indicate survival of the lower transverse anastomotic channel (Nagashima et al. 2010).

To the best of our knowledge, this is the first case of a patient who underwent pacemaker implantation via a RAIV. For cardiologists performing transvenous pacemaker insertion, the anomalous brachiocephalic vein may cause technical difficulty during a left arm approach. In cases in which left subclavian venography shows a tortuous vein that runs similar to the PLSVC but turns rightward before reaching the LA and connecting to the SVC, cardiologists should consider the presence of a RAIV. If it is too difficult to implant a pacemaker because of severe tortuosity, cardiologists need to consider implanting a pacemaker from the right side.

## Conclusions

A RAIV is a rare anatomical malformation that requires no treatment, but implantation of a pacemaker via a RAIV may be difficult. Thus, in cases in which subclavian venography shows a tortuous vein, cardiologists should consider the presence of a RAIV.

## Abbreviations

LIV: left innominate vein
RAIV: retroaortic innominate vein
SVC: superior vena cava
PLSVC: persistent left superior vena cava
RA: right atrium
LA: left atrium
